# The Story of the Hospitals

**Published:** 1887-05-28

**Authors:** 


### The Story of the Hospitals.

(*Continued from p. 124, vol. ii.*)

### The London Hospitals.

More Knowledge wanted.

Of the dozen or more great hospitals which exist in this metropolis how little is known by the average Londoner! Of the work which these institutions daily perform, and of the manner in which that work is done, still less information exists. Familiarity in this case has not bred contempt, but inacquaintance has induced indifference. People who are ready enough to grumble when an extra penny is put upon the Income Tax, or when the local assessment is raised one per cent., would be astounded could they realise what enormous sums are saved to them in their capacity as ratepayers by the hospitals. Human nature, after all, is prone to take the gifts which the gods provide without troubling itself overmuch to consider the sources whence the good things come, or the possibility of the stream some day running dry. Still, it must be confessed that the English people, at least, are not insensible to the claims of charity, provided their interest in the objects of their benevolence can be aroused and sustained; and if the hospitals of London are suffering from lack of support, it is because the public has only a most casual acquaintance with their methods of operation, and because no means exist—save the annually recurring Saturday and Sunday collections—by which the wants of the hospitals can be persistently brought under its notice. It is a fact, in the contemplation of which we of the Anglo-Saxon race may justly experience a feeling of pride, that only in English-speaking countries have secular hospitals, maintained entirely by voluntary effort, been established. Wherever we go they are sure, sooner or later, and generally sooner than later, to be found, monuments to the benevolence and the public spirit of our people. In the United States, in Canada, in Australia, and in our African colonies, hospitals exist on the same basis as amongst ourselves; whereas in the Latin countries hospitals are either associated with conventual and monastic foundations, or are established and directly supported by the State or the municipal authorities. Japan is trying very hard to recast her institutions on the English model, and to Anglicise her people as much as possible; but characteristics which are the development of centuries cannot be imported like parliaments and courts of judicature, so Tokio, the capital of the empire, has had to fall back upon the continental method in the matter of hospitals. Two have been established there, one by the State for the purpose of training native doctors, and another, a smaller one, by the municipality for the use of the poor. England, and especially London, being, as we have shown, so favourably situated in this respect, and the hospitals having been established and maintained without a penny of cost to the public exchequer—for the State discourages them, rather than otherwise, by allowing them to be taxed up to their full value, both for local and imperial purposes—prudence, not to speak for the moment of loftier considerations, obviously dictates their continued support on a scale which shall enable them to meet the demands of an ever-increasing population. The best, if not the only means of attaining this end, is to disseminate an intimate knowledge of their work, and by so doing to arouse a permanent and popular interest in their well-being.

The Causes of Hospital Poverty.

The efforts which have been made in this direction in recent years, and especially the holding, in connection with this year's Hospital Sunday collection in every district in the metropolis, of meetings in aid of that movement, would, on the face of it, seem to imply that there is now a lamentable falling off in the amount of public subscriptions as compared with former periods. The inference, however, would not be altogether correct. Undoubtedly, if the contributions to the hospitals of London be compared with the total population, the average per head of the inhabitants is miserably small; but the actual sums now collected are, as a matter of fact, larger now than ever they were, and the existing poverty of most of the hospitals is due rather to two causes—first, the largely increased cost of maintenance; and secondly, in the case of those institutions whose income is mainly derived from ancient endowments, the remarkable falling off in the value of land. As to the latter of these two causes, no demonstration is necessary in these columns; but as to the former, as being a matter less generally understood, some few facts by way of proof may usefully be cited. Just as the standard of living amongst all classes in the country has been enormously raised during the last forty years, so it has been with the hospitals. One of the oldest hospital superintendents in London declares that within his own experience, which embraces the period of forty years we have mentioned, the increase has been nearly a hundred per cent. The greater regard which is paid to sanitation; the revolution—for no other word is adequate to express it—which has taken place in the nursing professions; the more liberal prescription of what may be called the luxuries of life to patients; the vast strides which have been made in the art of surgery, by reason of which many cases which were formerly practically hopeless, are now the objects of careful operation and lengthy treatment; the greater attention now bestowed upon the wards, in order that by their bright and cheerful appearance they may tend to raise the spirits and promote the recovery of the patients;—all these, and many other causes, have contributed to swell the annual cost per bed at every hospital in the country. In a workhouse the average annual cost of each inmate is, roughly speaking, about eighteen guineas a year, and in the pauper infirmaries the expenditure does not much exceed £30; but in the London hospitals the cost per occupied bed varies from £70 to £110. And the reason of the discrepancy is plain. The duty of the parochial authorities, as custodians of the ratepayers, is to reduce the cost of maintenance to the lowest point consistent with the healthy existence of the paupers; but the aim of the hospitals is to cure, and to the end that a weak and suffering man may be made whole and returned to the world a useful member of society, a supporter of his family, and a source of wealth to the community, no reasonable expense is spared.

**The Old Order Changeth.** It would be interesting to compare the diets in an old hospital—Guy's or St. Bartholomew's, for instance—to-day and forty years ago; the frequency with which fowls, ice, champagne, and other expensive luxuries are now ordered would astonish those who have no practical acquaintance with the subject. Then, again, look at the difference in the nursing, and the considerable extra charge which this difference involves. Sairey Gamp and Mrs. Harris were, it may be said, exaggerations due to the exuberant fancy of a writer who sacrificed strict portraiture for the sake of broad and striking type of character. However that may be, nobody will deny the enormous change for the better which has taken place. And how has this change affected the hospitals? "Forty years ago," said the experienced gentleman to whom we have referred, "our nurses used not to be above scrubbing a floor, now we have another class of women who do that sort of work and nothing else, the ward-maids." Of course, the patients are proportionately benefited. With a sister as superior officer in each ward, an experienced, gentle nurse at hand by day and by night whose duties are strictly confined to the care of her charge, the bedridden sufferer of to-day can hardly realise what were the experiences of his predecessors a generation ago. It is within the mark to say that where one nurse was employed then two are employed now, in addition to the subordinate staff to which we have alluded. But besides this direct increase of cost there has been an indirect increase. With a ward-sister drawn from the upper middle ranks of life, and nurses whose station is oftentimes not much inferior, there have been necessitated better appointments and the provision of appliances for the comfort of the patients, all of which were undreamed of under the former *régime*.

**Why the Death-rate is Higher.** And yet, it is said, with all this extra expense, with all these accessories for promoting the ease and well-being of the occupants of the wards, the death-rate in the hospitals is not lower, but higher than it used to be. This, too, is true; and, like the other point, is easily explainable. With the growth of population the demands upon the hospital beds have correspondingly increased. So far as central London is concerned, it should be remembered that hospital accommodation is very much the same now as it was half a century ago, whilst year by year the human hives outside have become thicker and thicker, and the houses, for want of space, have clustered themselves round the very hospital walls. In these circumstances there has been nothing for it but to exercise a very careful selection. The survival of the fittest in the natural world has its counterpart in the retention of the unfittest of the scores of people who daily apply for admission to the hospital wards. Now that only the very worst cases are treated within the hospitals, it is not surprising that the death-rate is somewhat higher than it used to be when sufferers from comparatively trivial ailments were freely admitted to all the benefits of the highest medical skill and the best nursing.

With these observations on hospitals in general, we approach the detailed consideration of some of the London institutions, in the hope that we may be able to make the actualities of hospital life familiar to the busy world outside, and bring home to the public mind and conscience the widespread benefits which our hospital system confers upon the community at large.

(*To be continued.*)

